# Long Covid − The illness narratives

**DOI:** 10.1016/j.socscimed.2021.114326

**Published:** 2021-08-19

**Authors:** Alex Rushforth, Emma Ladds, Sietse Wieringa, Sharon Taylor, Laiba Husain, Trisha Greenhalgh

**Affiliations:** a Nuffield Department of Primary Care Health Sciences, University of Oxford, Oxford, OX2 6GG, UK; b Central and North West London NHS Foundation Trust, United Kingdom; c Imperial College School of Medicine, London, United Kingdom

**Keywords:** Long Covid, Post-acute Covid-19 syndrome, Covid-19, Narrative medicine, Online communities, Health social movements

## Abstract

Callard and Perego depict long Covid as the first illness to be defined by patients who came together on social media. Responding to their call to address why patients were so effective in making long Covid visible and igniting action to improve its care, we use narrative inquiry − a field of research that investigates the place and power of stories and storytelling. We analyse a large dataset of narrative interviews and focus groups with 114 people with long Covid (45 of whom were healthcare professionals) from the United Kingdom, drawing on socio-narratology (Frank), therapeutic emplotment (Mattingly) and polyphonia (Bakhtin). We describe how story-telling devices including chronology, metaphor, characterisation, suspense and imagination were used to create persuasive accounts of a strange and frightening new condition that was beset with setbacks and overlooked or dismissed by health professionals. The most unique feature of long Covid narratives (in most but not all cases) was the absence, for various pandemic-related reasons, of a professional witness to them. Instead of sharing their narratives in therapeutic dialogue with their own clinician, people struggled with a fragmented inner monologue before finding an empathetic audience and other resonant narratives in the online community. Individually, the stories seemed to make little sense. Collectively, they provided a rich description of the diverse manifestations of a grave new illness, a shared account of rejection by the healthcare system, and a powerful call for action to fix the broken story. Evolving from individual narrative postings to collective narrative drama, long Covid communities challenged the prevailing model of Covid-19 as a short-lived respiratory illness which invariably delivers a classic triad of symptoms; undertook and published peer-reviewed research to substantiate its diverse and protracted manifestations; and gained positions as experts by experience on guideline development groups and policy taskforces.

## 1 Introduction

In May 2020, two months after the Covid-19 pandemic was declared, the British Medical Journal published a patient blog (Garner, 2020). The author, a doctor himself, described still feeling ‘weird as hell’ six weeks after contracting suspected Covid-19, despite official advice stating that the typical recovery period for ‘mild’ (by which was meant non-hospitalized) cases was around two weeks. His was not the first account of Covid-19’s persisting symptoms, but it was probably the first to be picked up by the medical and mainstream press. Soon after, numerous similar stories appeared in mainstream media, social media and podcasts, describing the lived experience of more prolonged Covid-19. Patients described many, varied and fluctuating symptoms persisting beyond 3 weeks, coining the expression ‘long Covid’ to cover them all. Clinical recognition came somewhat later (Greenhalgh et al., 2020a).

Callard and Perego (2020) call long Covid a ‘patient-made illness’, the first to emerge by patients finding one another through social media. They describe how, in the absence of biomedical knowledge or medical legitimacy, online communities of Covid-19 ‘long haulers’ exchanged experiences and campaigned to raise awareness, influence guidelines and service models, and contribute to research.

In this paper, we explore long Covid’s rapid emergence and unique status among illnesses, using a dataset of narrative interviews and focus groups with people with long Covid. In a previous paper, we reported *what* these people talked about (Ladds et al., 2020b), here we analyse *how, why, and to whom* they told their stories (Kleinman, 1988).

## 2 Illness narratives in the context of COVID-19

Bruner (1990) argued that all experience is narratively structured. A narrative (story) is characterised by an unfolding of events and actions over time (chronology), a context or setting (scene), something going awry (trouble), characters (people of greater or lesser virtue who get in and out of trouble), and the literary devices such as surprise, suspense, and metaphor which convey meaning, motive, and causality (emplotment).

In the illness narrative, the ‘trouble’ is illness and the associated biographical disruption (Corbin and Strauss, 1988); the patient may be both victim (tragically struck by it) and hero (bravely bearing it). Bury (2001) distinguished contingent narratives (relating to the causes and immediate impacts of the illness), moral narratives (relating to the sick person’s social identity and moral status), and core narratives (which link sickness and suffering with deeper cultural levels of meaning). Frank divided illness narratives into restitution (where the illness gets better), quest (where the storyteller attains higher purpose through illness) and chaos (where the story doesn’t make sense until it has gained coherence through dialogue) (Frank, 1995), though this somewhat dated taxonomy has its contemporary critics (Gammelgaard, 2019; Woods, 2014). Much illness-related storytelling is oriented to making sense of the trouble by answering questions like “why me?”, “what caused it?”, “what can I do to get better?” (Kleinman, 1988). Told to an audience, illness narratives recruit empathic witnesses and inspire morally-motivated action (Kleinman, 1988).

Stories are told to particular audiences for particular purposes − usually with the intention of persuading.

“People do not tell their stories in a vacuum. They must fight (be good rhetoricians or debaters) to tell their story and to have it more or less accepted, authorized, or taken up by others. They try to control the circumstances of its hearing and, to some degree, of its interpretation.” (Kirmayer, 2000, 173)

As Bakhtin (1984) observed, all stories are a dialogue, since everything that is said happens in response to (or in anticipation of) a reaction from the listener or reader. Bakhtin introduced the term ‘polyphonia’ to convey that an unfolding group narrative is made up of multiple voices, each shaped in relation to the others.

Online support groups for long Covid illustrate the rhetorical and polyphonic nature of narratives. An additional feature of these groups, and one particularly worthy of exploration, is that *dialogue about this new illness occurred almost exclusively among patients* (Callard and Perego, 2020). Traditional work on illness narratives portrays the clinician as a therapeutic witness who, through actively listening to the patient’s story, helps them make sense of their illness and construct a healing narrative, helped by continuity of the clinical relationship over time (Balint, 1955; Frank, 1998). Attending to a patient’s narrative can enrich the diagnostic process (Greenhalgh, 1999), and strengthen, through moral imagination, their professional commitment to the patient (Charon, 2008).

Such therapeutic dialogue did not commonly occur with long Covid patients − for several reasons. First, the highly contagious nature of Covid-19 discouraged traditional face-to-face consultations, so algorithm-based triage and telephone callbacks replaced rich clinical dialogue. Second, during the study period (April to October 2020), the system under pressure prioritised deteriorating and potentially life-threatening conditions over symptoms that had become long-term. Third, as noted above, initial guidance suggested that Covid-19 was a short-lived illness, which meant that clinicians were not attuned to its more protracted effects. Finally, scarcity of diagnostic testing meant that few patients had had their acute Covid-19 illness confirmed, so there was no clear starting-point for the trajectory into long Covid.

For all these reasons, and with a few exceptions described below, long Covid was not only a new illness but one which, perhaps uniquely in recent history, emerged largely without the patient’s clinician acting as witness or sounding-board. In a book on narrative ethics in medicine, Rita Charon observed that “*The effective practice of medicine requires narrative competence, that is the ability to acknowledge, absorb, interpret and act on the stories and plights of others*” (Charon, 2001). The story of long Covid as it unfolded in 2020 includes an overarching meta-narrative of absent listeners: the collective failure − arguably for good reasons − of clinicians to acknowledge, interpret or act on their patients’ stories and plights.

As Charon has commented, the traditional therapeutic dialogue embraces both trust (the patient makes themself vulnerable through telling their story) and obligation (the clinician incurs ethical duties through hearing it) (2008). Long Covid calls for socio-narratological examination as a novel illness where both trust (on the patients’ part) and obligation (on their doctors’) went awry. Such a lens, moreover, may help explore the meta-narrative twist in long Covid’s own story − that doctors and health care workers were disproportionately affected (since Covid-19 is an occupational disease); for these people (45 of 114 in our sample), the subjective experience of long Covid and its management was informed by their clinical training and system knowledge.

The absent clinical witness in early long Covid narratives also contrasts with the many examples in the literature of illness narratives—including HIV, chronic fatigue and related syndromes, and some mental health conditions—in which the clinical witness has been depicted negatively as unsympathetic, disbelieving and wedded to a different model (Dumit, 2006; Foster, 2016; Schermuly et al., 2021; Woods et al., 2019).

### 2.1 Analytic framework and research questions

Drawing on theoretical concepts described above, we framed our analysis around the following features of long Covid narratives:

#### Trouble

we explored prolonged symptoms as the unwanted surprise in narrators’ lives (where had they come from?; what was at stake because of them?) and how they attempted to restore coherence.

#### Chronology

we analysed long Covid as a fluctuating and disabling illness with an uncertain time course, seeking to theorise how the Hei-deggerian concept of narrative time—and especially the future towards which the lived life is oriented (“*that place of desire where one is not where one wants to be, where one longs to be elsewhere*” (Mattingly, 2010, 124))—becomes disrupted.

#### Characters

we considered how illness narratives displayed and tested people’s character, and what kind of storytelling devices they used to depict themselves and others as heroes, victims, villains, innocent bystanders, clowns and so on. We extended Frank’s depiction of character (which mainly refers to individuals) to include how the characters of macro-actors and systems (e.g. the NHS or medical profession) were revealed and tested.

#### Emplotment

we considered how participants used literary devices, especially metaphor, surprise (twists in the plot) and suspense (“*the emotionally-charged moment of not-knowing*” (Mattingly, 1998)) to engage their audience in the tension. We explored the use of genres such as tragedy, which rely on listeners’ capacity as social beings to recognise contingency − how things might have worked out differently.

#### Veracity

we were interested in why and by whom the long Covid narratives had been believed and disbelieved. We studied stories *about* the narratives − in particular, narrators’ accounts of thwarted attempts to tell their stories and recruit health professionals to engage with, and act on, them.

#### Polyphonia

we approached our focus group data in particular as a multi-voiced narrative illustrating multiple perspectives (Bakhtin, 1984). We considered whose points of view were foregrounded and whose remained peripheral or silenced, and how different points of view came to influence subsequent stories.

#### Imagination

we considered how stories worked to arouse people’s imaginations, making aspects of lived experience visible and compelling and requiring listeners to recognise the fragility of events (the past might have turned out differently). We were especially interested in what Charon (2008) has called *moral* imagination in the context of clinical care.

#### Performativity

we studied narrative as performance, considering how storytellers sought to influence immediate and potential future listeners (or readers), drawing them into collective action through shared purpose. Using the emplotment devices described above, narrators propose certain narrative courses for listeners to enact collectively, thereby bringing about a better future (Frank, 2010; Mattingly, 2010).

These concepts provided a guiding framework with which to approach long Covid illness narratives, both individual and collective. In applying them, we were aiming not to achieve generalisability or representativeness, but to draw out resonance − how and why the stories and storytelling took the forms they did, how they affected their audiences, and to what future eventualities narrators were oriented.

In particular, we sought to address the following questions:

- What kinds of stories did people with long Covid tell − and what did they seek to achieve by telling them?
- How did storytelling inform and inspire new individual and collective identities and collective action?
- How did the absence (in most cass) of the storyteller’s clinician as therapeutic witness influence both individual narratives and the collective response?

## 3 Data collection and methods

### 3.1 Ethics and governance

Ethical approval was granted from East Midlands − Leicester Central Research Ethics Committee (IRAS Project ID: 283196; REC ref 20/EM0128) on 4^th^ May 2020 and subsequent amendments. The study was overseen by an independent advisory group with patient representation and a lay chair which met 3-monthly via video link.

### 3.2 Data collection and sampling

After an exploratory phase in April 2020, data collection occurred between May and October 2020. A dataset of 30 preliminary interviews was collected and transcribed in May and June 2020, before long Covid was widely publicised. Our initial aim was to interview people who had had suspected acute Covid-19 about their symptoms and illness journeys through the UK National Health Service (NHS). Recruitment occurred by social media requests via Twitter and snowballing from these primary contacts. One recruitment tweet from this early sample was posted on a Facebook support group for people with what we now know as long Covid, resulting in 20 people coming forward, keen to tell their stories of prolonged symptoms. We therefore designed the current sub-study, using theoretical sampling, to address not just the initial acute illness but also how it had unfolded subsequently.

We then recruited 10 additional participants via calls on social media and via long Covid support groups. To maximize variation, we tried (through explicit social media requests and targeted snowballing) to recruit from groups under-represented in our initial sample, including men, older people and people from black and minority ethnic groups. This led to 25 further individual interviews. One early finding was that health professionals were disproportionately represented in long Covid support groups (perhaps because of occupational risk), and that clinically trained participants had many ideas for improving long Covid services. For this reason, we extended our methodology to include focus groups where participants could discuss ideas for [re]designing services (Ladds et al., 2020a). Two focus groups were held with doctors with long Covid (n = 19), three with nurses and allied healthcare professionals with long Covid (n = 12), and three were with lay people (to gain a perspective uninfluenced by clinical training; n = 28).

Interviewers were clinicians (EL, SW, TG) and social scientists (AR, LH). Interviews lasted between 45 and 90 min and were narrative in format, inviting individuals to describe their early symptoms and what had happened to them since. Focus groups, all of which had one clinical and one social science facilitator, lasted 90 min and covered three broad topics − positive experiences with healthcare services, negative experiences, and ideas for improvement. In both cases, we used narrative prompts (such as “what happened next?”, “how did you feel about that?” or “can you describe that experience in a bit more detail?”).

All participants (n = 114) were invited to attend a 90-min Zoom presentation to feed back and comment on interim findings; 28 attended and contributed both verbally and via comments in the chat (with the option of posting anonymously).

### 3.3 Data analysis

Interviews and focus groups were professionally transcribed, anonymised and uploaded onto QSR NVivo 12 to support data familiarization, coding and analysis.

We undertook an initial thematic analysis (published elsewhere) of the symptomatic experience of long Covid (Ladds et al., 2020b) and ideas for improving services (Ladds et al., 2020a). When extending our analysis to consider stories and storytelling, we treated interview and focus group data more holistically as “*an evolving series of stories that are framed in and through interaction*”, and honed in on “*language use and other structural features of discourse*” (Reissman, 2012, 5). We sought to compare not just similarities and differences among participants’ stories but how they were told − especially how one narrative prompted both supporting narratives and counter-narratives in response.

Two authors theoretically coded sections of interview and focus group text in terms of the concepts outlined above. Coded sections of text were compared and discussed by the lead authors, then shared with other authors, who provided comments, criticisms and feedback on how to refine and rethink initial analysis. Two authors (AR and ST) brought their lived experience of long Covid to this analysis.

## 4 Findings

Participant characteristics are shown in Table 1. After a brief description of the online communities, findings are organised broadly around the core narrative themes described above. For each, we focus on how the act of storytelling enabled individuals to make sense of their own journeys (including coping with disappointment and overcoming setbacks), make their stories compelling to various audiences, and become part of a collective story and collective action.

### 4.1 The online communities

Participants were members of various online communities, summarised in Table 2. We have not given details of which groups participants belonged to so as to protect anonymity.

Different communities had different origins, vision and subcultures, but this multiplicity was primarily historical and their similarities were more significant than their differences. Relations between groups were cordial and had a strong sense of shared purpose. Some participants were members of several online groups; a few had been prompted to join by the Zoe Covid App, a widely-used smartphone application linked to a large UK research study, onto which people could enter symptoms (Menni et al., 2020). At least five (recruited to our study via social media or snowballing) were not members of any online groups.

### 4.2 A strange kind of trouble

A striking feature of the long Covid narratives was the unusual nature of the described symptoms. In early 2020, Covid-19 was depicted in clinical publications as a short-lived respiratory illness (often described as a ‘pneumonia’) with a defining triad of symptoms: fever, breathlessness and cough. Guidelines produced rapidly in early 2020 implied that unless patients met the criteria for hospital admission (usually with symptoms of pneumonia), they were likely to recover quickly and spontaneously (National Institute for Health and Clincial Excellence, 2020).

In contrast to this official narrative, long Covid as described vividly in the narratives was not short-lived, nor was it confined to the respiratory system. It also did not fit into a simple binary classification with severe cases hospitalized and mild ones managing unproblematically at home. While some participants had suffered a mild initial illness, our dataset also included some shocking accounts (which we have analysed in detail elsewhere (Greenhalgh et al., 2020b)) of people struggling alone with severe breathlessness, hypoxia that was documented on oximeters (“my sats were down to 88” − Participant FG AHG2) and exhaustion so profound they could barely move. Indeed, some people attributed their prolonged symptoms to the underdiagnosis and undertreatment of *severe* disease when they first became unwell.

Long Covid’s protean manifestations contrasted starkly with official emphasis on a clearly-demarcated defining triad. Symptoms were inconsistent and vague, affecting any or many body systems and came and went without rhyme or reason:

“I had an odd rash for quite a while it kept coming and going … very itchy cough … very mild asthma … I started getting the odd headache again …. Pins and needles feet going completely numb … all sorts of odd symptoms, I just kept putting it down to grief until a couple of months in a friend said, ‘Look do you think this could be Covid?’.” (Lay focus group participant FG1)

A common but not universal feature was cognitive blunting − which some participants called ‘brain fog’. The clinically-trained person in the quote below uses the metaphor of Windows and the Internet to depict how he lost the ability to think quickly and focus on multiple things at once:

“Two days ago I couldn’t remember the word brain. I described it as a thing that was like a blancmange in your head, the weirdest thing, these words just fall out of your head and I get very worried about going back to work. […] [N]ormally we [doctors] have got like about 12-odd Windows open and there’s about 12 different processes going on in our head, you’re seeing the patient there’s all sorts of things going on, you know, you’ve got other meetings with your staff, there’s messages popping in, you know … but at the moment I’ve got one Window open I can think about one thing at the time and half the time the Internet’s dodgy and it keeps blacking out.” (Doctor participant in FG1)

As illustrated in the opening paragraph of this paper and the above quotes, people with long Covid often used terms like ‘weird’, ‘strange’ and ‘odd’. The choice of these terms illustrates an important socio-narratological point: people were describing a pattern of symptoms which *did not make sense* in terms of existing lay models of illness − nor did they fit prevailing professional models of disease. As one of us has argued previously (Greenhalgh, 1999), the lay lexicon contains many terms that depict illness when it is *not* strange (‘poorly’, ‘not myself’, ‘washed out’), but when an illness is also depicted as strange, clinicians should be alert that the description *calls for explanation*. Unfortunately for patients, clinicians and researchers took some time to pick up on long Covid’s strangeness.

Many participants realised that their strange and persisting symptoms represented trouble only through prompting from others, including family or work colleagues. One or two described the Zoe Covid App’s promptings to consider long Covid. Narrators described their confusion lessening as others recounted similar experiences.

“It made me realise I wasn’t alone, and wasn’t losing my mind! As you mentioned, [government] symptom list [is] quite brief in comparison to the weird and wonderful things we all seem to be suffering with! To realise this was ‘normal’ was helpful” (Lay participant in chat forum during FG1)

### 4.3 Disrupted chronology and the cruelty of hope

Most people in our sample (106 of 114) had not been hospitalized. A prominent storyline for these participants was the trouble of failing to recover as predicted from what clinicians, had been describing as ‘mild’ Covid-19 (Callard, 2020).

Their stories of trouble drew on the literary device of the plot twist, which was depicted in one of four ways: 1) continuation of the acute phase of illness beyond the expected date of recovery (“my breathing and chest pain had really not improved at all, it was following this kind of daily cycle − worse at night time − but I was significantly impaired still at 5 weeks” − individual interview KT1); 2) feeling they were recovering or had recovered, but then experiencing a setback (“I’d had 11 days of feeling great. And after [a particular] weekend I crashed again. And again it seemed to last for weeks of having these waves of symptoms: shortness of breath, diarrhoea, muscle aches, complete fatigue.” − individual interview KS1); 3) a recurrent cycle of partial recovery followed by deterioration (“one day I’d feel not bad, then the day after I’d feel pretty terrible” − lay focus group participant HG1); or 4) as in the ‘odd rash’ quote in the previous section, initially assuming they were recovering, but later realising their strange and persisting symptoms could be due to the virus.

All these narratives were characterised by disrupted chronology: the trajectory of recovery in which they had been led to believe (an uncomplicated restitution narrative) was unexpectedly upset, and − given the uncertain prognosis − there was no indication of when it would be restored. Metaphors were telling: people talked of going ‘one step forward, one step back’, of ‘crashing’, and of being ‘stuck’. In all these storylines, the cruelty of hope axiom replays: there is desire, effort and anticipation towards a recovered future state, repeatedly dashed by lack of improvement, setbacks or the retrospective realisation that recovery has not even begun.

### 4.4 A doubting audience

The sense of confusion and dissonance resulting from long Covid’s disrupted chronology was heightened by the fact that many participants had not had formal clinical diagnosis of Covid-19 when they first became ill. Even fewer had received a positive swab test for Covid-19, since few people could access tests early in the pandemic and false negative results were common. Not only did participants find themselves overstaying their expected spell in (or repeatedly re-entering) the ‘kingdom of the sick’ (Sontag, 2001), but professionals, family, and even participants were questioning whether they had ever really entered (“Why didn’t I have antibodies, would anyone believe that I [had] had COVID?” Individual interview SN1).

The participants in our sample were thus not merely wounded storytellers (Frank, 1995) but failed rhetoricians (Kirmayer, 2000). Our dataset included an interesting sample of meta-stories (stories about storytelling), in which participants recounted to one another their accounts of describing their symptoms but being disbelieved or dismissed.

### 4.5 Frustrated becoming: a test of character

As Sayer has observed, “*A key characteristic of pain and suffering is that they are not merely states of being, but of frustrated becoming, or continuous yearning for relief and escape*” (Sayer, 2011, 42). Long Covid caused substantial disruption to the storytellers’ lives, as evidenced by their self-portrayal as previously healthy, fit and busy before the illness made it impossible to carry out even mundane tasks and routines, profoundly challenging their sense of self (Bury, 2001) and burdening them with the many self-care tasks needed to manage the illness’ impacts (Corbin and Strauss, 1988).

“…yesterday when I went to get my blood tests, I accidentally ended up getting five thousand steps on my step tracker because of the doctor’s trip and the pharmacy trip and, you know, moving around the house, getting dressed and stuff. It literally sent me crippled by six pm because it was just too much for me, compared to the days before where I’d done three thousand steps. So, even like the small increase like that just literally sends me completely washed out.” (Individual interview FC1)

These storylines were often accompanied by feelings of shame (towards themselves) or perceived blame (from others). It was evident that both the acute illness and − in particular − its longer-term sequelae provided a test of character for participants which they had been unable to meet heroically. The widely-held (but tragically misguided) belief that Covid-19 illnesses should clear at around two weeks contributed to a strong sense of stigma, which (Scambler, 2009, page 441) has defined as “*a social process, experienced or anticipated, characterized by exclusion, rejection, blame or devaluation that results from experience, perception or reasonable anticipation of an adverse social judgement about a person or group.*”

As they shared their narratives and discovered the common experience of both shame and blame, a collective sense of anger developed towards unjust treatment from a society that had prematurely defined what was ‘normal’ for this illness.

### 4.6 The healthcare system as a lottery

Stories about interactions with healthcare services were sometimes presented as a quest to be seen and cared for − evolving (occasionally) into a story of restitution or (more commonly) ongoing tragedy in which the narrator did not receive the assessment, tests and treatments that might have set them on a path to recovery.

Even where specialist services existed, not all clinicians seemed aware of them and referral pathways were exclusionary or non-existent. Participants described being ‘sent around the houses’ in a usually fruitless trail. In these narratives, the NHS was sometimes depicted as a mis-cast, stumbling character who had prepared for a different role (that of acute responder) and was unable to adapt to the very different challenges of long Covid. Narrators used words like ‘random’, ‘lottery’ and ‘luck’ when describing their attempts to navigate services. The gambling metaphor depicted both the rare existence of fit-for-purpose assessment and rehabilitation services and the fact that actually receiving such a service was (at the time of the study) unlikely. Several participants described paying for private care to avoid this ‘lottery’.

During the pandemic, the structures and communication channels for booking a traditional GP appointment had been replaced by an un-familiar and disorienting ‘remote-by-default’ service based on telephone, email, chatbot and video. The brain fog described above made navigating this technology-saturated terrain particularly challenging, especially since staff were still applying (and software had been built around) an acute respiratory illness model for Covid-19. Some people described how they learned to adapt their account of symptoms to fit triage algorithms designed before long Covid’s symptomatology was known.

In the later months of our data collection, after long Covid had been picked up by the media and received some dedicated funding for establishing specialist clinics, patients’ narratives shifted emphasis from the ‘lottery’ of accessing their GPs towards a new ‘lottery’ of trying to access specialist long Covid services. One focus group participant coined the term ‘unicorn clinics’. Rather than radically shifting the storyline, these clinics were framed as another chapter in an ill-fated quest to access the healthcare system.

### 4.7 Clinicians in the narratives: present and absent witnesses

Narrators’ clinicians (usually but not always doctors) were sometimes central characters in their narratives, depicted either positively or negatively. ‘Heroic’ status was sometimes linked to being willing to initiate investigations and referrals. More commonly, however, the hero clinician was the one who listened to, acknowledged and validated the narrator’s suffering − a role which, as Frank (1998) has emphasised, is crucial for patients experiencing deep (i.e. serious and life-changing) illness.

Some participants appreciated their doctor’s willingness to build a therapeutic alliance and be honest about the associated uncertainties. They valued the doctor’s efforts to accompany and guide them as they strove to acquire knowledge about this new condition and to help them cope with disappointment when the illness or system let them down.

“I think it was a really positive experience and I felt really listened to, and she was able to be honest at that point and said I don’t really know what I can do to help you but you can phone me or e-mail me at any point.” (Doctor participant in focus group FG1)

Schei (2006) has defined this combination of therapeutic continuity, empathy, and willingness to learn as the essence of clinical leadership. It was a recurring source of frustration and hurt in long Covid communities that many clinicians in the narratives were unable or unwilling to take on this role.

Many narrators were empathetic and forgiving towards their clinicians when contextual factors featured prominently (for example, when the disease was new, the system overwhelmed, or they were having to make difficult judgments via remote technology). However, they were less forgiving when clinicians’ professionalism appeared deficient − for example, when they refused to accept the legitimacy of the patient’s symptoms or the condition more generally; when they ‘fobbed off’ their patients (advising them, for example, to attend the Accident and Emergency Department rather than offering a consultation, or offering benzodiazepines for assumed anxiety); when they dismissed requests for tests even when these were perceived to be indicated (for example, cardiac tests in people with chest pain) and when they failed to provide therapeutic attention and active listening. Individual testimonies of such experiences gained credibility as others offered similar stories − and also as rare counter-narratives surfaced of doctors who *had* engaged both clinically and emotionally with their plight despite the pressures of the pandemic.

Gaslighting − deliberately psychologically tormenting and undermining the patient’s account of their illness − was mentioned in several interview and focus group accounts (“*I’m very, very angry with quite a few GPs because I felt like they gaslighted me quite significantly*” − participant in lay focus group FG1). Clinically-trained participants in our sample tended to be more knowledgeable of the pressures within the system and keen to avoid accusations of mismanagement or ‘gaslighting’ of patients by their doctors. Rather, they were keen to use their combined clinical and experiential knowledge to help redesign services—a topic we have covered elsewhere (Ladds et al., 2020a)).

### 4.8 The online community: collective witnessing and knowledge-sharing

Our individual interviewees commented that stories about negative experiences with health services and healthcare providers were frequently shared in their online support groups. In our focus groups, we observed directly the sharing, and empathic hearing, of such experiences. Bearing witness to others’ stories, re-telling these, and affirming the moral claims implicit in them, appeared to be an important aspect of belonging to a long Covid community. It helped members recognise their own suffering as patterned and legitimate, gave individual narratives a polyphonic quality (Bakhtin, 1984), and contributed to an emerging collective identity (Shostak and Fox, 2012).

The affirmation and empathy provided by the long Covid online groups had a dramatic impact on some members, shifting them from a chaos narrative to quest (Frank, 1995), as in this example:

“I managed to get onto the Facebook group and, and that was the beginning of me feeling a lot more comforted I suppose you could say because like at last, at last I knew that there were just thousands of people around the country and in fact in other countries as well that were being affected the same way as me … and they felt they weren’t being supported and it was through this group that you sort of suddenly, you feel a bit stronger and you say right I can, I can now perhaps go and find out a little bit more or I can … ask for some further investigations … so in the, in the next couple of weeks it was just like this support group were, were my salvation.” (Individual interview BE1)

In contrast to widespread (though by no means universal) rejection of patients’ accounts by clinicians, the online community was considered a safe space in which stories could be shared and believed. More practically, it was also a forum for exchanging knowledge about recovery practices (especially self-help treatments such as diet and nutritional supplements, and approaches to exercise and pacing), as well as discussing research.

Many commented that joining online support groups had made them more knowledgeable than the healthcare professionals with whom they were interacting − a situation which is known to generate awkward interactional dynamics in clinical consultations (Snow et al., 2013).

Members of online communities also shared suggestions of how to mobilize support from family and friends. Solidarity was evident as members who had access to a support network acknowledged the position of those who did not:

“I don’t know how I would have coped on my own, listening to [another participant] talk about living on her own […] at least I had somebody there to bring me food and drink and do the laundry.” (Allied health professional in focus group FG2)

As in the above example, narrators often pointed out that others had ‘had it worse’ than them, reflecting what Charmaz and Belgrave (2013) call an implicit ‘hierarchy of moral status’ in stories of suffering.

The echo-chamber nature of some online groups, which invariably attracted like-minded individuals, prompted some to leave. We inter-viewed one person, for example, who had had a spell in intensive care; they had left the group because few members had shared this experience. Others have described a similar feeling of ‘not belonging’ to a so-called support group (Locock and Brown, 2010). A group of doctors with long Covid set up their own group as they found what they termed ‘doctor bashing’ on some of the other support sites tiresome. Several of our interviewees (recruited through snowballing) were not members of online support groups and did not feel the need to join one.

### 4.9 Taking collective action

Some online communities became a springboard for action to establish long Covid as a legitimate disease in medicine, healthcare, and wider society − thus enacting a collective version of Frank’s (1995) quest narrative. Long Covid stories convey knowledge about a novel and complex condition − and impart moral lessons about what needs to be done to support people with it.

Long Covid illness narratives emplotted potential pathways of action for several audiences including fellow patients, clinicians, researchers and policymakers. Group organisers, for example, saw mobilising their members to contribute stories to our own study as a way to influence the official knowledge base and raise awareness of long Covid among clinicians (who, they felt, would then take patients more seriously). More generally, the groups sought to dispel myths, ensure that patients developing symptoms of long Covid would be forewarned with knowledge about the condition and the system, and use their lived experience to redesign services. Some became spokespeople for their groups, speaking on television and writing newspaper articles.

Some long Covid online groups collectively imagined, planned and undertook research. Some was biomedically-framed—for example, large symptom surveys of members published in peer-reviewed journals (Assaf et al., 2020; Goërtz et al., 2020). Some was more sociological and philosophical—for example, defending open-ended terminology that embraces rather than sidesteps the illness’s many uncertainties, and arguing for a democratisation of the research process (Perego et al., 2020).

## 5 Discussion

### 5.1 Summary of main findings

This study of the illness narratives of 114 participants has described how people with long Covid came together in online communities and used storytelling devices (including chronology, metaphor, characterisation, suspense and imagination) to create persuasive accounts of a novel illness journey that was beset with setbacks and often overlooked or dismissed by health professionals. Their narratives were striking in terms of the strange and unpredictable nature of the symptoms, the extent and duration of disrupted chronology, the narrator’s experience of the healthcare system as a ‘lottery’, and the absence (often for pandemic-related reasons) of a professional witness to many of the stories. Instead of sharing their narratives in therapeutic dialogue with their own clinician, people struggled with a fragmented inner monologue before finding an empathetic audience and other resonant narratives in the online community. Individually, the stories seemed to make little sense, but collectively they provided a rich description of the diverse manifestations of a grave new illness, a shared account of rejection by the healthcare system, and a powerful call for action to fix the broken story. Evolving from individual narrative postings to collective narrative drama, long Covid communities challenged the prevailing model of Covid-19 as a short-lived respiratory illness which invariably delivers a classic triad of symptoms; and undertook and published peer-reviewed research to substantiate its diverse and pro-tracted manifestations. Stories were (and continue to be) a crucial resource for long Covid sufferers to build awareness and mobilize action in others.

Situated against the backdrop of a global pandemic, long Covid experiences ran counter to stories circulating about the nature and threat of Covid-19, including some early accounts of scientific experts and government scientists (who suggested, for example, that only certain categories of people were at risk of serious harm; that unless hospitalized, those infected will only experience a brief respiratory illness; and that ‘herd immunity’ was a scientifically and ethically defensible strategy).

### 5.2 Strengths and limitations of the study

A significant strength of this study is the large sample size with diversity in ethnicity, gender, and age (Table 1). Our sample, whilst not demographically representative of the general population, was (through oversampling of particular subgroups) more so than the membership of long Covid online communities, which are predominantly young or middle-aged, white and female (Assaf et al., 2020). Whereas all our participants were from UK, long Covid online communities are international, though predominantly from USA and UK.

One key limitation of our sample was that we recruited all but 7 participants via social media (n = 28) or online groups (n = 79), so we had limited data on the experiences of the less digitally connected. Notably, the one interviewee in our sample who had had no experience at all of social media or online groups was particularly confused and frustrated; their account had the classic features of a chaos narrative. Our lack of evident gender or ethnic differences in illness experience may have been due to the relatively small numbers of men and non-White participants in our sample. Another limitation was that we did not observe the interactional dynamics of the online communities directly. We chose not to do so because it felt disrespectful to the mission of the groups. Indeed, the study began when one member of one group spotted a posting from one of us on Twitter and approached *us* with the suggestion that we should include long Covid patients in our interviews. We felt that if they had wanted us to observe their online community, they would have invited us. Instead, we changed our study design to include focus groups so we could observe and capture some interactions among the participants. Some commented that they enjoyed meeting their online ‘Covid buddies’ in a video group setting and having their first verbal interaction. Nevertheless, not being in the online groups ourselves meant that narrative exchanges occurring in these groups did not make it into our dataset except as re-told tales.

Some aspects of the study were potentially both strengths and limitations. The sampling strategy meant that many narratives had been honed and even become stylised within online communities before being shared in a research context. On the one hand, this meant that some stories were ‘fully formed’ and confidently related; on the other hand, aspects of individual experience which resonated with a wider group narrative may have been over-emphasised. The high proportion of clinicians in both the research team and the participants themselves meant that many narratives would have been constructed for a [partially] clinical audience. However, the interviews collected by non-clinical researchers covered very similar themes and were not obviously less medicalized.

### 5.3 Comparison with other illness narrative research

Sociological research on illness narratives can be divided broadly into three types. First, the autobiographical monologue as exemplified by Frank’s (1995) classic story of testicular cancer and heart disease as a young man. Second, research undertaken by clinicians or social scientists who interview a purposive sample of patients about their illness, with or without an ethnographic component − for example, chronic pain (Werner et al., 2004) or chronic fatigue (Whitehead, 2006). A number of findings in such studies resonate with our own current study: an illness which the narrator assumed would be short-lived became protracted; a mismatch between the illness experience and societal and medical expectations which generated shame, blame and confusion; unsuccessful efforts by the narrator to be a ‘good patient’ and return to health through personal effort; health professionals depicted as disbelieving, unsympathetic and dismissive; and (in most cases) the narrative becoming stuck in chaos or tragedy genre. A third type of illness narrative research, discussed below, focuses on storytelling in online communities.

It is important to acknowledge challenges to the prevailing narrative turn. Woods (2011), drawing on critical scholars (Gabriel, 2004; Strawson, 2004), warns that narrative analysis may uncritically valorize the individual story, brush aside the complex relationship between narrative and truth, overlook the potential for the illness narrative to damage as well as heal, tend to misclassify any qualitative account of experience as a “narrative”, fail to attend to genre, and generate acultural and ahistorical elements of individual stories—leading, Woods suggests, to an over-emphasis on the “agentic, authentic, autonomous storyteller” and individual identity. Elsewhere, she critiques the power-charged way in which patients’ “recovery narratives” can become circumscribed, misappropriated and even fetishized by clinicians and policymakers in mental health (Woods et al., 2019).

Acknowledging such critiques, we agree that our study design-—which involved researching *with* rather than *on* the people whose stories formed our dataset—formed a very particular context of inquiry in which—as Gabriel (2004) puts it—“*storytellers and audiences are bound by a psychological contract which regulates legitimate and non-legitimate forms of representation or ‘regim*”. In other words, in respecting our participants’ stories and seeking to capture them authentically, we had limited scope to challenge their veracity (though that was never our goal). Furthermore, the interpretation presented in this paper does not rest on a reified or exclusionist view of narrative. Our starting assumption—that ill people tell and share stories and that both the stories themselves and the process of story-sharing are worth studying—is entirely compatible with Woods’ and others’ claims that narrative doesn’t explain everything and that other literary forms are also worth studying (Gammelgaard, 2019; Woods, 2011, 2014). Our study of collective story-sharing illustrates that a focus on identity work by the lone storyteller is not the only way of applying narrative concepts to accounts of illness.

### 5.4 Comparison with research on online patient communities

Ziebland and Wyke (2012) reviewed a disparate literature and identified seven mechanisms through which online communities helped people live with illness: finding information, feeling supported, main-taining relationships with others, influencing behaviour, experiencing health services, visualizing disease and learning to tell one’s story—of which the last is particularly relevant to our findings. Foster (2016) studied message board interactions in a prominent online community and affirmed the role of the community in shaping individual and collective narratives and exchanging tacit knowledge about how to navigate the system. The title of his article—’Keep complaining till someone listens’—illustrates the powerful role some of communities play in promoting self-advocacy within a [perceived] hostile system.

Kingod et al.’s qualitative systematic review (2017) summarised the findings of 13 primary studies of online patient communities. They found four activities common to most: illness-associated identity work (often with emotionally-laden disclosure and drawing support from peers), social support and connectivity (overcoming isolation through new friendships and addressing how to handle altered family relations), experiential knowledge sharing (particularly about the practicalities of living with illness, complementing the more disease-based knowledge imparted in clinics), and collective voice and mobilization (especially advocating for changes in health services). They also found that members of these communities found them valuable and empowering, and that early concerns raised by clinicians that these communities would be dangerous sources of misinformation appeared largely unfounded. Studies published since that systematic review include Kingod’s own empirical study (2020) of diabetes online communities which introduced the actor-network theory derived concept of ‘tinkering’ (exchanging practical strategies for coping), and Hargreaves et al.’s study (2018) of ‘sharing and empathy in digital spaces’ by people with breast cancer and motor neuron disease. Similar impacts of oral story-telling (meaning-making, empathy, exchange of practical wisdom, support for navigating the healthcare system, and collective action) were demonstrated in face-to-face peer support groups (Greenhalgh et al., 2011).

Whilst online patient communities are often experienced by their members as supportive and empowering, a recent study questions whether the self-advocacy and activism made possible through them is always in patients’ best interests (Schermuly et al., 2021). An alternative (Foucauldian) interpretation is that they represent a form of governmentality, in which the patient, through the community, becomes “responsibilized” to self-manage their condition in “agentic” ways that shift responsibility from State to citizen. As Schermuly et al. point out, long Covid online communities appear to be less “agentic” and more “activist” than some (e.g. breast cancer), perhaps as a result of the pressing need to advocate for the very existence of the illness and establishment of basic services for it (Callard and Perego, 2020).

## 6 Conclusion

This study has affirmed long Covid as a patient-defined illness which gained legitimacy largely through the stories and actions of online communities. Using a socio-narratology lens, we surfaced how these groups achieved therapeutic emplotment and a strong collective identity by the telling, re-telling and affirmation of stories.

Since we completed our data collection (October 2020), long Covid patient communities in the UK have earned a place at the policy table, with their members invited to join national guideline groups and task forces as ‘experts by experience’. While some people with long Covid remain unwell with no clear path to recovery, accounts of full and partial recovery are beginning to appear. It will be interesting to revisit the long Covid narratives in the future to see what new dialogues are occurring between those who have recovered and those who have not.

## Figures and Tables

**Table 1:** Participant characteristics.

	Individual interviews	Focus group participants	Total
Total participants	55	59	114
Age			
• Median	48	43	46
• Range	31−68	27−73	27−73
Ethnicity			
• White British	39	45	84
• White other	4	4	8
• Black	3	2	5
• Asian	7	6	13
• Mixed	2	2	4
Clinical training			
• Doctor	3	19	22
• Nurse or allied health professional	11	12	23
• Non-clinical (layperson)	41	28	69
Recruited from			
• Online patient group	31	48	79
• Social media	17	11	28
• Other	7	0	7
Hospital experience			
• Admitted to hospital	3 [1 ITU]	5 [1 ITU]	8 [2 ITU]
• Seen in A&E but not admitted	7	16	23
• Did not go to hospital	45	38	83

ITU = intensive therapy unit.

**Table 2:** Examples of Long Covid online communities.

Name (url)	Origin, membership and main focus
Long Covid Support longcovid.org	Established by colleagues in UK and Italy; now one of the largest online groups. Provides support and resources for people with long Covid who were not admitted to hospital. Links to sister groups in several countries. ∼21,000 members
LongCovidSOS longcovidsos.org	“The voice of thousands of long Covid sufferers in the UK’. Campaigning for more and better research. Organised an open letter to the UK Prime Minster and campaigned for patient representation on guideline groups and policy bodies. ∼18,000 members.
Body Politic wearebodypolitic.org	TOriginally established in the US by friends who supported each other through early stages of long Covid. Insipred Long COVID SOS and other Covid-19 patient advocacy campaigns. “We wanted to provide others with that same opportunity to feel connected and supported through infection, symptoms, and recovery’. Seeks to “[break] down barriers to patient-driven whole-person care and well-being, particularly for historically marginalized communities”. ∼25,000 members
Positive path of wellness (Facebook group previously known as CovidUKLongHaulers)	“A group purely to support each other and gather information on all of our experiences and journeys”. UK-based. ∼3000 members
UK Doctors’ Long Covid Facebook group	“A group for UK doctors to share emerging data about long term Covid symptoms and a space for support for doctors experiencing symptoms.” ∼1000 members, though not all have long Covid (some are doctors who seek to learn from others’ lived experience).
Covid-19 Survivors Group UK https://www.selfhelp.org.uk/COVID-19_Survivors_Group_UK	Branch of Self Help UK. “A new service for people who have had coronavirus and survived”. ∼1300 members.
